# Effect of Compaction Pressure on the Enzymatic Activity of Pancreatin in Directly Compressible Formulations

**DOI:** 10.3390/pharmaceutics15092224

**Published:** 2023-08-29

**Authors:** Daniel Zakowiecki, Peter Edinger, Tobias Hess, Jadwiga Paszkowska, Marcela Staniszewska, Svitlana Romanova, Grzegorz Garbacz

**Affiliations:** 1 Chemische Fabrik Budenheim KG, Rheinstrasse 27, 55257 Budenheim, Germanytobias.hess@budenheim.com (T.H.); 2 Physiolution Polska sp. z o.o., Skarbowcow 81/7, 53-025 Wroclaw, Polandg.garbacz@physiolution.pl (G.G.); 3 Department of Pharmacognosy, National University of Pharmacy, Pushkinska 53, 61002 Kharkiv, Ukraine; 4 Physiolution GmbH, Walther-Rathenau-Strasse 49a, 17489 Greifswald, Germany

**Keywords:** pancreatin, protease, amylase, enzymatic activity, calcium phosphate, direct compression

## Abstract

Tableting of biomolecules is a challenging formulation phase due to their sensitivity to various process parameters, such as compression pressure, process dynamics, or the temperature generated. In the present study, pancreatin was employed as a model enzyme mixture, which was formulated in tablet form utilizing the synergistic effects of brittle and plastic excipients (dibasic calcium phosphate and microcrystalline cellulose, respectively). The effect of varying compaction pressure and lubricant concentration on the generated temperature and enzymatic activity was evaluated. The tablets were analyzed for pancreatin content and the activity of two enzymes (protease and amylase) using pharmacopoeial tests. This study indicated that the formulations proposed here allow tableting over a wide range of compaction pressures without adversely affecting pancreatin content and its enzymatic activity.

## 1. Introduction

In recent years, enzymes have found widespread use and are of growing interest in modern medicine. The industrial market for such drugs is expected to grow rapidly in the coming years. Enzymes are selective biocatalysts of high efficiency present in living organisms. In many cases, their enzymatic activity depends on the pH of the environment and temperature. Regarding the latter factor, it should be taken into consideration that as proteins, they can denature at higher temperatures and lose their functionality. In this regard, the optimal temperature can vary from enzyme to enzyme [1,2,3].

Oral solid dosage forms (OSDFs) are still the most preferred option by both drug manufacturers and patients. Among them, tablets are the most widely and willingly used [4]. Nevertheless, the technological processes employed in the production of tablets can significantly decrease enzymatic activity. The safest are dry processes, which do not pose a risk of enzyme decomposition during processing in the presence of water and/or during the following drying [5,6,7]. Therefore, manufacturing of tablets using the direct compression method (DC) seems to be the most appropriate formulation strategy. Nevertheless, the compression forces (compaction pressures) applied during the tableting process can be a key factor potentially affecting enzyme stability [5,8,9]. Schulz et al. revealed that compaction pressure induced a reduction in powder volume, which was the main cause of the loss of enzymatic activity of butyrylcholine esterase and peroxidase during tableting [10]. Similarly, Teng and Groves observed a decrease in enzyme activity during compression of urease [11], and Wurster and Ternik found the same in the case of catalase [8].

It has been reported that the type and nature of excipients incorporated into formulations can affect enzyme stability. Picker found that enzyme inactivation can be mitigated by the employment of excipients that require low compaction pressure and are able to re-lease mechanical stresses during tableting [12]. Graf et al. showed that the use of micro-crystalline cellulose exerted a stabilizing effect on enzymes in pancreatin tablets [13]. However, Kuny and Leuenberger as well as Sharma et al. noticed that at higher concentrations, MCC can induce high shear forces and cause the enzyme particles to be crushed [14,15]. In their study, Sharma et al. linked the loss of model enzyme activity to a decrease in porosity and an associated increase in tablet density under applied compression forces. This was explained by the collapse of voids (pores) in compressed powders leading to mechanical damage of protein molecules [15]. It should be noted that enzyme powders are not identical and differ in terms of their mechanical properties and deformation characteristics. Consequently, it is important to employ the matching excipients providing a good balance between plasticity and brittleness. This will ensure that the compressed enzyme tablets will have sufficient mechanical properties and the required performance without losing the activity of the active ingredient.

The subject matter of the presented research was pancreatin, which is a dry extract of fresh animal pancreas. It contains a combination of several digestive enzymes with amylolytic, proteolytic, and lipolytic activities that are activated in the alkaline pH in the small intestine [16,17]. Pancreatin has been used for many years to treat digestive disorders caused by insufficient secretion of pancreatic enzymes. In addition, pancreatin supplementation has long been widely used by athletes [18,19]. Commercial products are marketed as dietary supplements, over-the-counter (OTC) and prescription (Rx) medications, in the form of enteric-coated powders, granules, capsules, or tablets [20,21].

Marketed products are characterized by a fairly wide variety of enzyme compositions and activities, which very often do not match those declared. Löhr et al. analyzed several commercial formulations of pancreatic enzymes in terms of their enzyme activities [22]. They found significant variation from the declared contents, which for lipase changed from 93% to 115%, for amylase from 97% to 233%, and for protease from 120% to 281%. Maev et al. determined the strengths of the commercial pancreatin products by determining lipase activity [23]. Determined enzyme activities expressed as a percentage of labeled lipase activity in various products ranged from 79% to 122%. A similar approach was presented by Kuhn et al. [24] and Shrikhande et al. [25]. The products tested differed significantly in the percentage relationship between labelled and assayed lipase activity, which ranged from 102% to 187% in the former case, and from 55% to 120% in the latter.

The aim of the present study was to develop directly compressible formulations of pancreatin. Furthermore, to investigate how factors acting on compressed powders affect the pancreatin content and its enzymatic activity. The study also examined the impact of the compression force (compaction pressure) or the tableting speed affected the characteristics of the tablets (density, hardness, friability, disintegration). The analyzed factors additionally included ejection forces and the temperature generated during the compression process. The latter parameter was analyzed with the help of a thermal imaging camera.

The synergistic action of two excipients showing a different deformation mechanism, i.e., plastic microcrystalline cellulose and brittle calcium phosphates, was utilized in the formulation design. Such a combination gives powder mixtures the desired flowability and allows the preparation of tablets with good mechanical strength, rapid disintegration, and fast release of a drug substance [26]. Moreover, the application of highly porous grades of calcium phosphates in the formulation allows the matrix of the tablets to maintain an adequate level of porosity, preventing a strong reduction in volume during com-pression and preventing damage to the enzyme molecules [27].

## 2. Materials and Methods

Pancreatin (from porcine pancreas) EMPROVE^®^ ESSENTIAL USP was from Merck KGaA (Darmstadt, Germany). Anhydrous dibasic calcium phosphate, DCPA (DI-CAFOS^®^ A150), tribasic calcium phosphates, TCPs (TRI-CAFOS^®^ 500 and TRI-CAFOS^®^ 200-7) were produced by Chemische Fabrik Budenheim KG (Budenheim, Germany). Low-substituted hydroxypropyl cellulose, L-HPC (LH-11) was provided by ShinEtsu (Wiesbaden, Germany). Microcrystalline cellulose, MCC (VIVAPUR^®^ 102 was from JRS Pharma (Rosenberg, Germany), Magnesium stearate (Ligamed^®^ MF-2-V) was supplied by Peter Greven Fett-Chemi (Venlo, The Netherlands).

### 2.1. Pancreatin Tablet Formulations

Two formulations with the qualitative and quantitative compositions given in Table 1 were tested in this study. In the first formulation, DCPA was used as the main filler/diluent and in the second one, TCP. The final tableting mixtures were prepared by mixing pancreatin with all excipients except the lubricant in a Turbula^®^ mixer (Willy A. Bachofen AG, Muttenz, Switzerland) at 36 rpm for 20 min. After the addition of the lubricant, the powder mixtures were blended at the same speed for a further 5 min. The obtained tableting mixtures were analyzed in terms of their flow characteristics using the angle of repose (AoR) method according to the description given in chapter 2.9.36. “Powder flow” of Ph. Eur. 10.0.

The final blends were compressed into tablets of the weights shown in Table 1 with increasing compression forces of 10 kN, 15 kN, 20 kN, and 25 kN (corresponding to 157 MPa, 236 MPa, 314 MPa, and 393 MPa, respectively) using a Fette 102i rotary tablet press (Fette Compacting GmbH, Schwarzenbek, Germany) equipped with concave punches of 9 mm diameter (Fette Compacting GmbH, Schwarzenbek, Germany) and operated at two press speeds, i.e., 31.3 rpm and 62.5 rpm. The highest compression force recommended by the punches manufacturer was 25 kN.

This study also analyzed the effect of reduced lubricant concentration on the ejection forces and temperature of the ejected tablets. For this purpose, the lubricant concentration was reduced to 0.5% and 0.25% compared to the formulations listed in Table 1. These changes were compensated by the amount of the main filler/diluent so as to maintain the final weight of the tablets.

### 2.2. Analysis of the Tablets

The temperature of the compressed tablets was measured immediately after they were ejected from the tablet press dies with the aid of an infrared thermal imaging camera Fluke Ti32 (Fluke Europe B.V., Eindhoven, The Netherlands) equipped with a 320 × 240 pixels Focal Plane Array Detector. Images were captured at a refresh frequency rate of 9 Hz and analyzed with a thermal imaging software SmartView^®^ version 4.3.311.0 (Fluke Thermography, Everett, WA, USA).

Tablets hardness (expressed as tensile strength), weight, and thickness were analyzed using a Semi-Automatic Tablet Testing System SmartTest 50 (Sotax AG, Aesch, Switzerland) and averages were calculated based on the analysis of 10 randomly selected tablets.

Friability was tested with the friability tester Friabilator (USP) EF-2 (Electrolab, Mumbai, India). The number of tablets corresponding to 6.5 g were weighed and tested at a speed of 25 rpm for 4 min. The tablets were weighed again, and the mass was compared with their initial weight.

A disintegration test was carried out with an SDx-01 disintegration tester (Secom GmbH, Hamburg, Germany) in 900 mL of purified water at the temperature of 37 °C. Disintegration times of 6 individual tablets were recorded.

### 2.3. Assay of Pancreatin

The determination of pancreatin content in the developed formulations was performed with a UV-Vis Spectrophotometer Agilent 8453 (Hewlett Packard, Palo Alto, CA, USA) equipped with a 10 mm quartz cell at 280 nm. Sample solutions were prepared by weighing 100 mg of crushed tablets into 50 mL volumetric flasks, mixing vigorously with phosphate buffer, then placing in a PURA 22 water bath (JULABO GmbH, Seelbach, Germany) at 40 °C for 10 min, and cooling. Samples were prepared in triplicate. Prior to analysis, they were centrifuged using a bench-top Centrifuge 5417 R (Eppendorf AG, Hamburg, Germany) at 14,000 rpm for 15 min, and the clear supernatant was collected for measurement.

The phosphate buffer was prepared by mixing 51 mL of a monobasic potassium phosphate solution (13.6 g/500 mL of water) with 49 mL of a dibasic sodium phosphate solution (14.2 g/500 mL of water). The pH of the solution was checked using an 8603 SevenMulti pH meter (Mettler-Toledo AG, Schwerzenbach, Switzerland) and adjusted to 6.8 with a sodium hydroxide solution. The buffer was prepared daily.

A calibration curve was prepared by making a series of solutions over a range of pancreatin concentrations from 0.4 to 1.2 mg/mL. For this purpose, appropriate amounts of pancreatin standard were weighed into 25-mL volumetric flasks and proceeded as for the preparation of test solutions. The determined correlation coefficient R2 was 0.9990. The content of pancreatin in tablets was calculated as a percentage in relation to its theoretical content, i.e., 150 mg (see Table 1).

### 2.4. Analysis of Digestive Enzyme Activity

The activity of pancreatin in the developed formulations was determined for two enzymes: amylase and protease. Both analyses were carried out according to the procedure described in the USP 44–NF 39 monograph for pancreatin tablets and the referenced monograph for pancreatin, with minor modifications concerning preparation of standard and sample solutions. The final concentration of the solutions was adjusted based on the theoretical activity of pancreatin, which was used in the formulations, as well as its content in the tablet final blends. This amounted to 25 mg of pancreatin standard in 25 mL of pH 6.8 phosphate buffer as per the USP 44–NF 39 monograph for pancreatin and, correspondingly to the developed formulations, 100 mg of crushed tablets in 50 mL of the same buffer. Both standard and sample solutions for the two enzymes tested were prepared in the same way as for the pancreatin content determination.

The pharmacopoeial method for assessing amylase activity is based on evaluating its ability to digest starch. After the enzymatic reaction, the amount of undigested starch is determined by titrating its complex with iodide. The enzymatic reaction was carried out as per the USP 44–NF 39 monograph for pancreatin under “Assay for amylase activity (Starch digestive power)”. From the results of the titration with sodium thiosulphate, the amylolytic activity of pancreatin was calculated using the equation from the aforesaid monograph.

The pharmacopoeial method for assessing protease activity is based on evaluating its ability to digest casein. Casein, which is used as a substrate, undergoes proteolysis during the enzymatic reaction. The degree of proteolysis is determined spectrophotometrically by measuring the absorption of aromatic amino acids present in digested casein fragments. The enzymatic reaction was carried out as per the USP 44–NF 39 monograph for pancreatin under “Assay for protease activity (Casein digestive power)”. Spectroscopic analysis was carried out using a UV-Vis Spectrophotometer Agilent 8453 (Hewlett Packard, Palo Alto, CA, USA) equipped with a 10 mm quartz cell at 280 nm. Protease activity was calculated by comparing the absorbance values obtained for the standard solution and the sample solution, as described in the referenced monograph.

## 3. Results

### 3.1. Characterization of Pancreatin Formulations

The investigated pancreatin powder was characterized by poor flowability—AoR of 47.7 ± 0.5 degrees. In the DCPA formulation (see Table 1), there was a need for an additional glidant, which improved the flow characteristic to a “fair” level as per Pharmacopoeia (AoR 37.7 ± 1.1 degrees) and enabled non-problematic direct compression. Of the initially tested glidants, the most efficient was fine calcium phosphate (TRI-CAFOS^®^ 200-7), which was incorporated into the final formulation. In the case of the TCP formulation (see Table 1), the directly compressible grade of tribasic phosphate (TRI-CAFOS^®^ 500) was able to improve the flow characteristics to such a level (AoR 32.9 ± 0.5 degrees, which according to Pharmacopoeia, is described as “good”) that the additional use of a glidant was not necessary.

**Figure 1:**
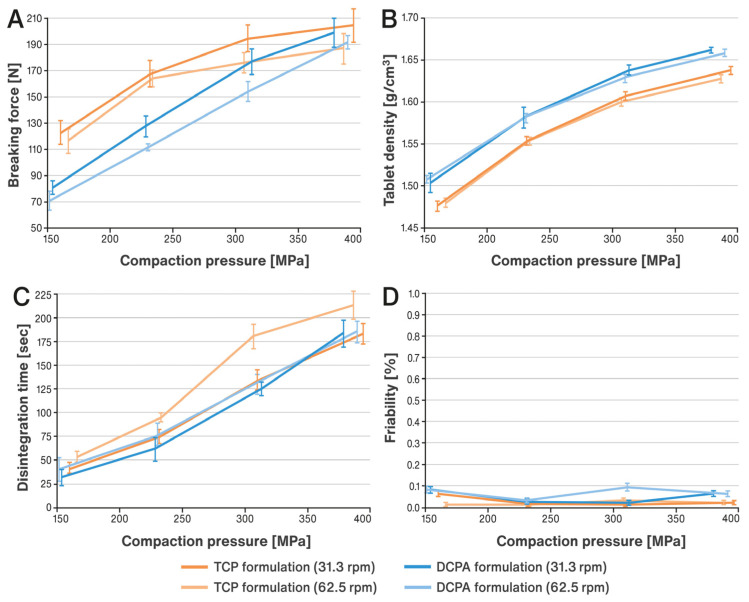
Effect of compaction pressure on pancreatin tablet characteristics: (**A**) hardness expressed as breaking force (mean of n = 10), (**B**) density (mean of n = 10), (**C**) disintegration time (mean of n = 6), (**D**) friability (mean of n = 3); SDs are indicated by the error bars.

Both formulations with the compositions given in Table 1 were compressed into tablets containing 150 mg of pancreatin using increasing compression forces (compaction pressures). The obtained tablets were analyzed for their physical properties as described in the Materials and Methods section. The results are illustrated in Figure 1A–D.

### 3.2. Effect of Process and Formulation Variables on Ejection Force and Tablet Temperature

**Figure 2:**
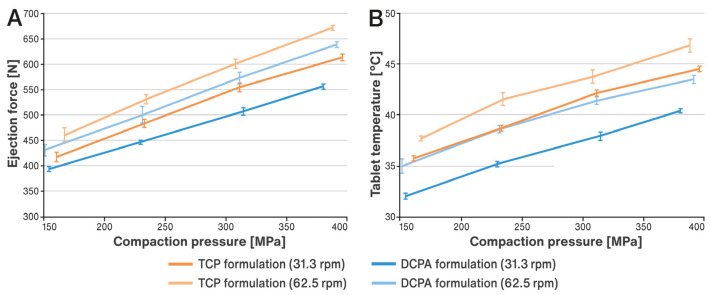
Changes in ejection force (**A**) and tablet temperature (**B**) induced by increasing compaction pressure.

**Figure 3:**
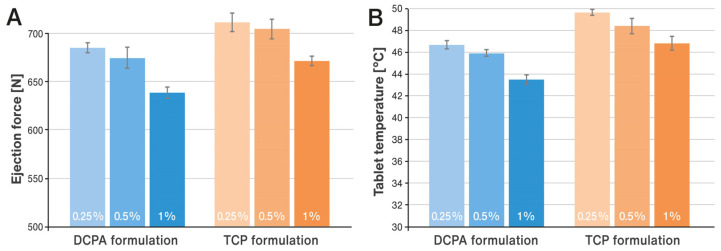
Changes in ejection force (**A**) and tablet temperature (**B**) caused by a reduction in the concentration of lubricant in the formulation (indicated inside each bar).

**Figure 4:**
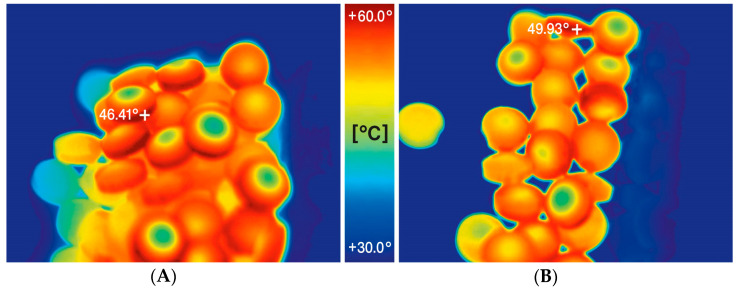
Exemplary measurement results processed by the thermal imaging software for DCPA formulation containing 0.25% of lubricant compressed with a force of 25 kN (**A**) and TCP formulation containing 0.25% of lubricant compressed with a force of 25 kN (**B**) with maximum observed temperature displayed.

The impact of increasing compaction pressure on the ejection forces and temperature of the tablets is illustrated in Figure 2A,B. Additionally, Figure 3A,B show how these parameters are affected by decreasing lubricant concentration in the formulation. The results shown in Figure 2B and Figure 3B represent the temperature of the tablets measured on their surface immediately after being ejected from the tablet press die using an infrared thermal imaging camera. Exemplary results of measurements processed by the thermal imaging software are shown in Figure 4A,B.

### 3.3. Evaluation of Pancreatin Content and Enzymatic Activity in Compressed Tablets

**Figure 5:**
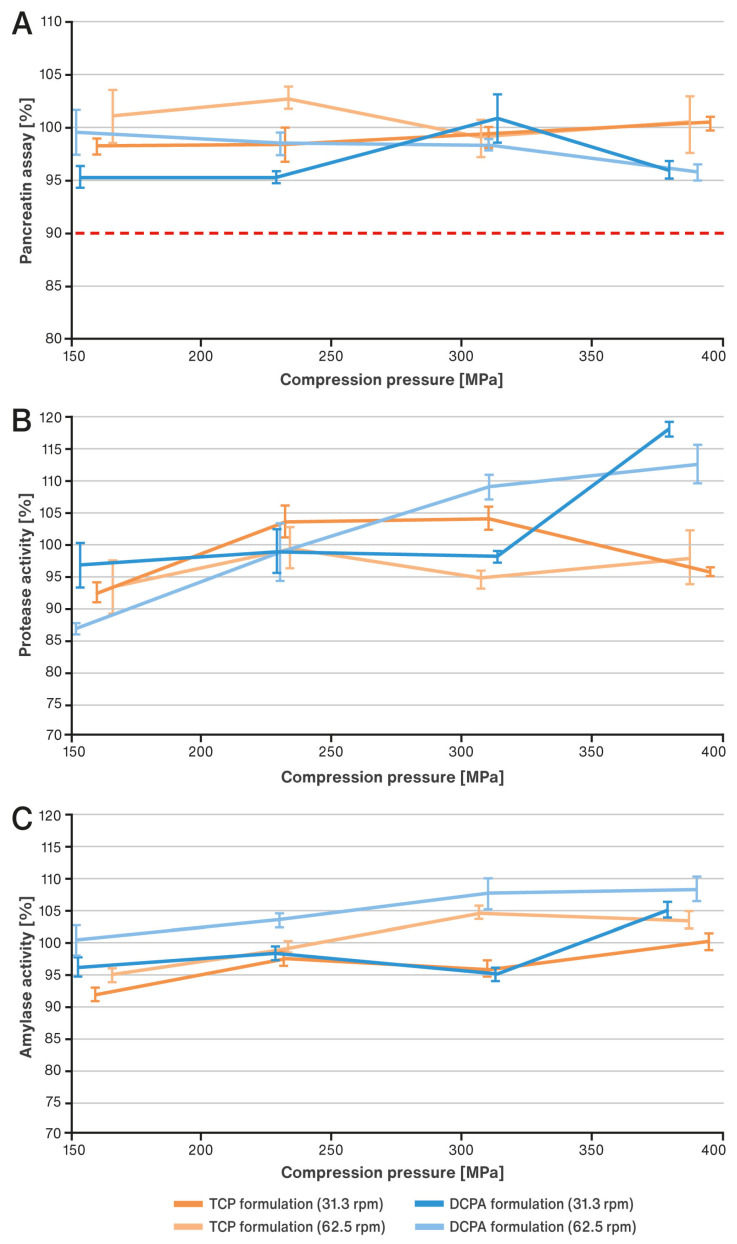
Impact of compaction pressure on pancreatin content (**A**), enzymatic activity of protease (**B**) and amylase (**C**). The dotted line indicates the limit required by the USP 44–NF 39 monograph for pancreatin tablets: not less than 90.0 percent of the labeled amount of pancreatin.

**Figure 6:**
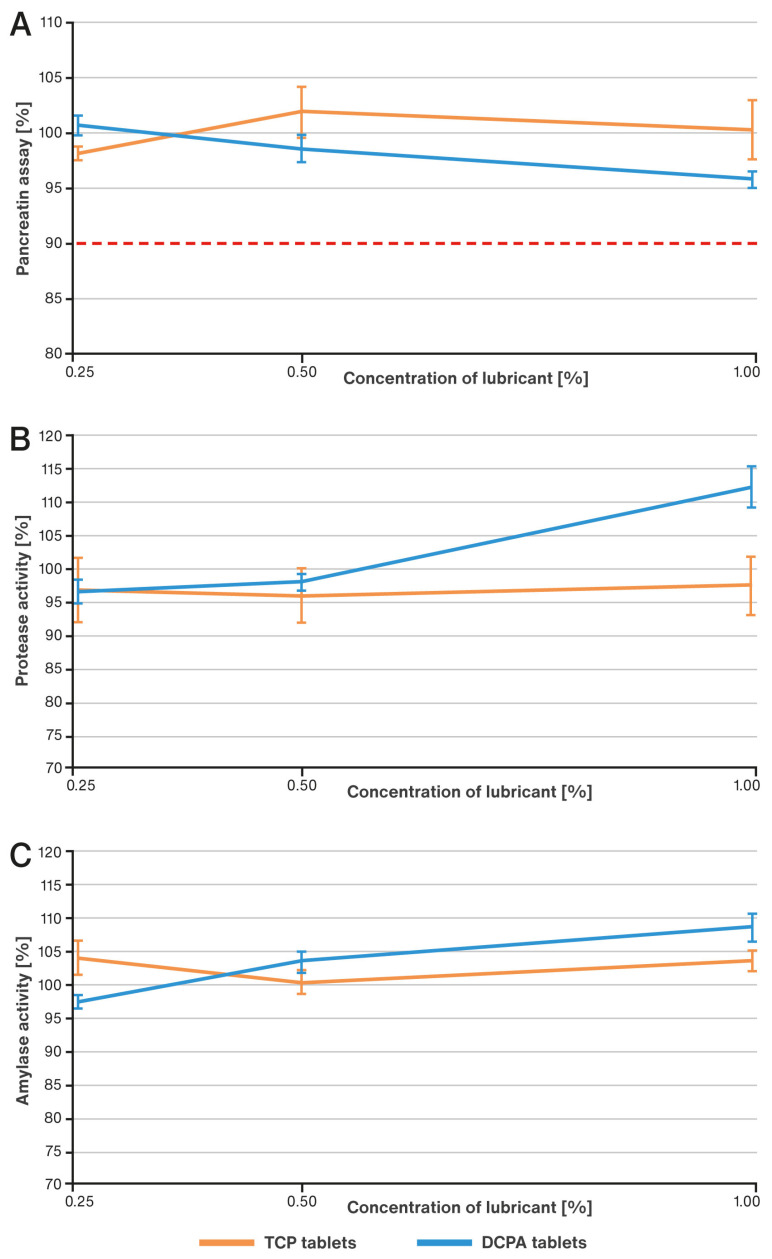
Effect of decreasing concentration of lubricant on pancreatin content (**A**), enzymatic activity of protease (**B**) and amylase (**C**). The dotted line indicates the limit required by the USP 44–NF 39 monograph for pancreatin tablets: not less than 90.0 percent of the labeled amount of pancreatin.

The effect of increasing compaction pressure on the content of pancreatin in the developed tablets as well as enzymatic activity, based on the examples of protease and amylase, is illustrated in Figure 5A–C. Analogously, changes in the same characteristics under the influence of decreasing lubricant concentration are shown in Figure 6A–C. The results of the active substance content are presented in percentages in relation to its theoretical content in the tablet. The results of enzyme activity expressed in percentages refer to the initial activity in the tablet final blends (before compression).

## 4. Discussion

This work aimed at the investigation of process variables such as compaction pressure or tableting speed on pancreatin content and its enzymatic activity in DC tablet formulations. The design of these formulations exploited the synergistic effects of two popular excipients widely used in pharmaceutical technology as fillers/diluents and exhibiting different deformation characteristics. Microcrystalline cellulose was chosen as the plastic component and calcium phosphate as the brittle one [26]. Regarding calcium phosphate, two types employed in the study, i.e., DCPA (DI-CAFOS^®^ A150) and TCP (TRI-CAFOS^®^ 500), were characterized by high porosity and large specific surface area (23 m^2^/g and 85 m^2^/g, respectively) [28]. These properties were supposed to facilitate obtaining tablets with adequate mechanical strength, while protecting them from excessive densification, which was reported to be one of the factors responsible for enzyme inactivation [14,15].

The pancreatin used in the study showed poor flowability, which was considered a basic technological problem to be solved, as it could hinder DC process. In the case of the DCPA formulation, the addition of a glidant was necessary to give the tableting mixture the required flowability and allow its DC. A 1% *w*/*w* admixture of fine grade of TCP (type TRI-CAFOS^®^ 200-7) allowed for trouble-free tableting with the help of a rotary tablet press equipped with a force feeder. At the same time, in the case of TCP formulation, it was observed that the coarse grade of tribasic calcium phosphate employed (type TRI-CAFOS^®^ 500) was able to improve the flowability of tableting mixture so significantly that there was no need for additional application of a glidant. It also allowed reducing the number of DC formulation components to only five. Both developed tableting mixtures, i.e., the one based on DCPA and the second based on TCP, were compressed with increasing forces, while monitoring the resulting ejection force and surface temperature of the produced tablets.

**Figure 7:**
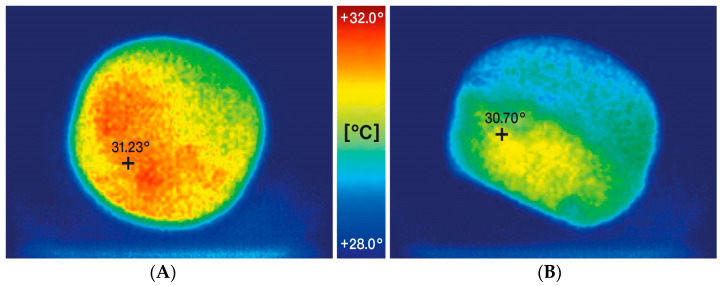
Comparison of the temperature of a tablet (DCPA formulation) compressed at 10 kN compression force measured on the surface immediately after ejection from the die (**A**) and inside after breaking (**B**); the maximum measured temperature in the first case reached 31.23 °C, in the second 30.70 °C.

The latter aspect is extremely important because, as previously reported, pancreatin, being an enzyme, can be inactivated at temperatures too high [1,2] and tableting is a process that generates large amounts of heat energy [29,30,31]. To monitor this factor, the study employed a thermal imaging camera, which allowed recording the maximum surface temperatures of the tablets immediately after they were ejected from the tablet press die. A similar approach was successfully applied in earlier studies by Ketolainen et al. [32]. At the beginning of the experiments, the temperature on the surface of the tablet and inside it after breaking was compared (see Figure 7). The observed temperature inside was lower than on the surface by less than one degree Celsius. It can, therefore, be assumed that the measurement of the maximum temperature on the surface of the tablet reflects well the temperature shock to which the tablet is subjected during the tableting process.

It was observed that for both formulations investigated, the increase in ejection forces resulting from the use of higher compaction pressures was accompanied by an almost linear growth in tablet temperature (see Figure 2). Ejection forces were not particularly high and did not exceed 700 N (Figure 2A); similarly, growing temperatures remained at a moderate level and did not exceed 50 °C (Figure 2B). The ejection forces and correlated tablet temperatures generated during compression were higher for the TCP formulation than for the DCPA formulation. Tableting at a faster speed (62.5 rpm) produced a greater increase in tablet temperatures in both cases. The effects described above did not cause significant changes in the content of pancreatin in the tablets produced. All results met the requirements of the USP 44–NF 39 monograph for pancreatin tablets, i.e., they contained more than 90.0% of the labeled amount of the active ingredient. Assayed contents did not form any clear trends and ranged approximately from 95% to 103% (see Figure 5A).

Analyzing the results of enzymatic activity of amylase and protease, they seem to show a slight upward trend with increasing compression pressures and associated thermal effects. One can notice a quite evident effect of the speed of tableting (see Figure 5B,C). The results of amylase activity in tablets compressed at a faster speed, i.e., 62.5 rpm, are higher than those prepared at a lower speed of 31.3 rpm for both tested formulations. When considering only the thermal effect, this would suggest a favorable effect of a mild increase in temperature on the enzymatic activity, especially of amylase. Nevertheless, it should be noted that the activity is higher for the DCPA formulation, which generated lower ejection forces and associated temperatures than the TCP formulation. Thus, the temperature of the tablets seems not to be the sole relevant factor. For the second enzyme, protease, there is also an upward trend in the results of this enzyme’s activity with increasing compaction pressure/temperature, but only for the DCPA formulation. For the TCP formulation, it is difficult to establish a clear trend in the results.

The effect of processing conditions was examined for the formulations described in Table 1, which contained 1% *w*/*w* of lubricant. As shown in the study, such a concentration prevented excessive increases in ejection forces and tablet temperatures. In the next step, the effect of decreasing concentration of the lubricant on the above-mentioned parameters was analyzed. For this purpose, the content of magnesium stearate was reduced to 0.5% and 0.25% in relation to the compositions listed in Table 1. This change was compensated for by a corresponding increase in the content of the main filler, i.e., DCPA or TCP, so as to maintain a constant tablet mass. The tablets were prepared at the highest compression forces allowed for the punches used (25 kN, corresponding to approximately 393 MPa) and at a higher rotational speed, i.e., 62.5 rpm. For both formulations, an increase in ejection forces and associated tablet temperature was observed as the concentration of the lube decreased (see Figure 3A,B). For the TCP-based formulation, the observed values were always measurably higher than for the DCPA formulation. No effect of the concentration of the lubricant was observed on the content of pancreatin in the tablets, which ranged from about 95 to 102% (see Figure 6A). Analysis of the enzymatic activity of amylase and protease shows an increase in the results for the DCPA formulation. In the case of the TCP formulation, no clear trend of changes in enzyme activity was noticed (see Figure 6B,C).

The results indicate that for both tablet formulations, the content of pancreatin as well as its enzymatic activity determined on the basis of amylase and protease remained relatively stable. However, a slight increase in enzymatic activity could be observed under conditions promoting a small rise of temperature (not exceeding 50 °C). In this regard, both the DCPA- and TCP-based formulations have shown good performance, even at reduced concentrations of lubricant (see Figure 6). Nevertheless, under the conditions of routine pharmaceutical production, the mechanical strength of the tablets is an important aspect, ensuring that they can withstand the mechanical stress of subsequent stages of the production process, such as coating, sorting, or packaging [33,34]. Therefore, the tablets prepared in this study were analyzed for hardness, friability, or disintegration time. Tablet density was also an important consideration, as it can affect enzymatic activity [15].

TCP formulation showed very good tableting properties, and even at the lowest compression forces of 10 kN (compaction pressure of about 150 MPa), the hardness of tablets expressed in terms of breaking force markedly exceeded 100 kN and friability did not exceed 0.1% (see Figure 1A,D). In the case of the DCPA formulation, one can clearly notice its brittle nature and the almost linear relationship between compression forces and tablet hardness. At higher applied compression forces, the tablets have a very similar hardness to the TCP formulation. The friability of tablets of DCPA formulation is slightly higher, but still does not exceed 0.12%. It is also evident that the slower tableting speed (31.3 rpm) facilitated obtaining tablets with better mechanical properties (i.e., hardness and friability). The compression of the powders resulted in a significant densification/reduction in volume for both formulations examined. The tablet densities of the DCPA formulation were 1.5–1.9% higher than that of the TCP formulation. In this case, no effect of tableting speed was observed (see Figure 1B). Despite significant densification during tableting, no associated decrease in enzymatic activity was observed. This reduction in volume was probably caused by pore collapse and did not result in mechanical damage to the pancreatin particles. At the same time, the loss of porosity did not radically prolong the disintegration time of the tablets. The disintegration times of the tablets of both formulations were very similar and increased as their hardness increased (see Figure 1C). It should be noted that even for the hardest tablets, the disintegration time did not exceed 4 min, which is many times shorter than the limit given in the USP 44–NF 39 monograph for pancreatin tablets, i.e., 60 min.

## 5. Conclusions

The application of excipients exhibiting different deformation mechanisms in the formulation of sensitive enzymes, such as pancreatin, resulted in a synergistic effect that enabled the successful preparation of compressed tablets which were safe for the active substance. The most favorable was the use of a combination of a brittle substance (anhydrous dibasic calcium phosphate) and a plastic one (microcrystalline cellulose) mixed in a weight ratio of 2:1. This allowed tableting over a fairly wide range of compression pressures without a significant increase in the ejection forces and associated temperature rise. In addition, the produced tablets showed very good mechanical properties and ensured the maintenance of adequate enzymatic stability, as demonstrated by the examples of protease and amylase.

It is also worth mentioning that other methods of drug delivery in the form of very small capsules are being developed, which allow for the complete omission of the compression procedure and may prove to be a safe solution for formulations containing sensitive enzymes in the future [35,36].

## Figures and Tables

**Table 1:** Qualitative and quantitative composition of pancreatin tablet formulations.

	DCPA Formulation		TCP Formulation		
Ingredient	mg	%	mg	%	Role
Pancreatin	150	46.66	150	47.14	Active
TRI-CAFOS^®^ 500	-	-	100	31.43	Filler/diluent
DI-CAFOS^®^ A150	100	31.10	-	-	Filler/diluent
VIVAPUR^®^ 102	50	15.55	50	15.71	Filler/diluent
L-HPC LH-11	15	4.67	15	4.71	Disintegrant
TRI-CAFOS^®^ 200-7	3.25	1.01	-	-	Glidant
Ligamed^®^ MF-2-V	3.25	1.01	3.2	1.01	Lubricant
Total	321.5	100.00	318.2	100.00	

## Data Availability

All data/results collected in this study are presented in the article.
